# Elevated expression of chloride intracellular channel 1 is correlated with poor prognosis in human gliomas

**DOI:** 10.1186/1756-9966-31-44

**Published:** 2012-05-11

**Authors:** Liang Wang, Shiming He, Yanyang TU, Peigang Ji, Jianhai Zong, Jingyu Zhang, Fuqiang Feng, Jipei Zhao, Yongsheng Zhang, Guodong Gao

**Affiliations:** 1 Department of neurosurgery, Tangdu hospital, Fourth Military Medical University of PLA, No.569, Xinsi Road, Baqiao District, Xi’an City, 710038, China; 2 Department of experimental surgery, Tangdu hospital, Fourth Military Medical University of PLA, No.569, Xinsi Road, Baqiao District, Xi’an City, 710038, China

**Keywords:** Chloride intracellular channel 1, Glioma, Real-time quantitative RT-PCR assay, Immunohistochemistry, Prognosis

## Abstract

**Background:**

Chloride intracellular channel 1 (CLIC1) is expressed ubiquitously in human tissues and is involved in the regulation of cell cycle, cell proliferation and differentiation. Recent studies have shown that CLIC1 is highly expressed in several human malignant tumors. However, its roles in human gliomas are still unclear. The aim of this study was to investigate the clinicopathological significance and prognostic value of CLIC1 expression in human gliomas.

**Methods:**

CLIC1 expression in human gliomas and nonneoplastic brain tissues was measured by real-time quantitative RT-PCR assay and immunohistochemistry. Its association with clinicopathological factors or prognosis in patients with gliomas was statistically analyzed.

**Results:**

The expression of CLIC1 at both mRNA and protein levels was significantly increased in high-grade (Grade III~IV) glioma tissues compared with that in low-grade (Grade I~II) and nonneoplastic brain tissues, and was up-regulated with ascending tumor World Health Organization (WHO) grades. The elevated expression of CLIC1 protein was also significantly correlated with low Karnofsky performance score (KPS) (P=0.008). Moreover, both univariate and multivariate analysis shown that high CLIC1 expression was significantly associated with poor prognosis in patients with gliomas (P<0.001 and P=0.01, respectively). In particular, the elevated CLIC1 expression also correlated with shorter overall survival in different glioma subgroups stratified according to the WHO grading.

**Conclusions:**

Our data provide the first evidence that CLIC1 expression might play an important role in the regulation of aggressiveness in human gliomas. The elevated expression of CLIC1 might represent a valuable prognostic marker for this disease.

## Introduction

Human gliomas represent the most common primary brain tumors in both children and adults. According to histopathological and clinical criteria established by the World Health Organization (WHO), this dismal disease can be classified as well-differentiated low grade astrocytomas [World Health Organization (WHO) grade I~II], anaplastic astrocytomas (WHO grade III) and glioblastoma multiforme (GBM, WHO grade IV) [1]. Despite recent therapeutic advances, the survival of patient with glioma is still poor. The median overall survival of patients with malignant gliomas is no more than one year and local recurrence occurs in more than 90% of patients [2]. Recent studies have indicated that patients’ age, Karnofsky performance status (KPS) score, histologic grade, and tumor necrosis are important prognostic factors for gliomas [3]. However, the prognosis of both high- and low-grade tumors remains heterogeneous. The median survival time of patients with high-grade gliomas range from 5 to 59 months and some patients with low-grade tumors also present poor outcome [4]. Similar with other human solid tumors, the predominant features of gliomas are extensive local tumor invasion and metastasis, in which multiple molecular events are involved. Focusing on these genetic background and molecular pathogenic processes is necessary to identify novel diagnostic and prognostic markers for improving the clinical outcome of patients with gliomas.

In mammals, the chloride intracellular channel (CLIC) gene family has six members, including CLIC1, CLIC2, CLIC3, CLIC4, CLIC5, and CLIC6 [5]. This family is defined by a conserved, approximately 230 amino acid core sequence which comprises the C-termini of all known CLICs. CLIC1 is a newly discovered member of the CLIC family [6]. In 1997, it was originally cloned from a human monocytic cell line activated by the phorbol ester, phorbol 12-myristate 13 acetate [7]. CLIC1 is expressed ubiquitously in human tissues and is usually localized in the cytoplasm and nucleoplasm with a soluble form. It has been demonstrated to be involved in the regulation of cell cycle, cell proliferation and differentiation [8]. In the G2/M phase, CLIC1 is detected on the plasma membranes of cells, and the inhibition of CLIC1 function prolongs the mean time of the cell cycle in cell culture [9]. Recent studies have found that CLIC1 is over-expressed in malignant tumors, such as hepatocellular carcinoma [10], gallbladder carcinoma [11], gastric carcinoma [12], and colorectal cancer [13,14]. CLIC1 has been considered as a sensor and an effector during oxidative stress, which may lead cells through all the phases of the cell cycle [15]. Thus, CLIC1 is hyperactivated in cancer cells which are in a highly proliferative state, and plays important roles in tumor invasion and metastasis.

In central nervous system, several lines of evidence support that CLIC1 plays a fundamental role in activated microglia and is involved in the pathophysiology of several neurodegenerative diseases [16]. Additionally, Kang et al. [17] found that small cell populations of GBM2 cancer stem cells (CSCs) were resistant to chemotherapeutic agent BCNU and highly expressed CLIC1. They further demonstrated that CLIC1 was involved in the resistance of BCNU-resistant CSCs. However, the clinicopathological significance and prognostic value of CLIC1 in clinical glioma specimens are still unclear. To address this problem, CLIC1 expression in human gliomas and nonneoplastic brain tissues were measured by immunohistochemistry. The association of CLIC1 immunostaining with clinicopathological factors or prognosis of glioma patients was statistically analyzed.

## Materials and methods

### Patients and tissue samples

This study was approved by the Research Ethics Committee of Tangdu Hospital, Fourth Military Medical University, P. R. China. Written informed consent was obtained from all of the patients. All specimens were handled and made anonymous according to the ethical and legal standards.

A total of 128 formalin-fixed, paraffin-embedded specimens of gliomas resected between 2000 and 2010 were retrieved from the archives of the Pathology Department of Tangdu Hospital, Fourth Military Medical University, P. R. China. All the slides were re-evaluated according to WHO classifications [1] by two pathologists, with differences resolved by careful discussion. A total of 76 males and 52 females (1.46:1) were enrolled in this study, and the median age was 42 years (range, 12–71). Thirty-two of the 128 gliomas were classified as low-grade [18 pilocytic astrocytomas (WHO I) and 14 diffuse astrocytomas (WHO II)], and 96 were classified as high-grade gliomas [38 anaplasia astrocytomas (WHO III), and 58 primary glioblastomas (WHO IV)]. None of the patients had received chemotherapy or radiotherapy prior to surgery. The clinicopathological features and the treatment strategies of all the patients were indicated in Table 1. Paraffin and snap-frozen sections of nonneoplastic brain tissues from 10 patients with intractable epilepsy were also included as controls.

**Table 1:** Clinicopathological features of 128 patients with gliomas

**Features**	**WHO I**	**WHO II**	**WHO III**	**WHO IV**
**Case No.**	18	14	38	58
**Mean age (year)**	38.6	45.9	43.1	44.2
**Gender**				
Male	12	6	25	33
Female	6	8	13	25
**KPS**				
>80	15	11	9	15
<80	3	3	29	43
**Surgery**				
Gross total resection	18	14	28	38
Partial resection	0	0	9	15
Biopsy	0	0	1	5
**Adjuvant treatment**				
Radiotherapy	0	0	30	12
Chemotherapy	0	1	0	6
Radiotherapy and Chemotherapy combination	0	0	5	28

In addition, 20 glioma specimens [5 pilocytic astrocytomas (WHO I), 3 diffuse astrocytomas (WHO II), 3 anaplasia astrocytomas (WHO III), and 9 primary glioblastomas (WHO IV)] were snap-frozen in liquid nitrogen and stored at −80°C following surgery for real-time quantitative RT-PCR assay.

Five years follow-up was performed, and all patients had complete follow-up until death. Overall survival time was calculated from the date of the initial surgical operation to death. Patients, who died of diseases not directly related to their gliomas or due to unexpected events, were excluded from this study.

### Immunohistochemistry assay

Formalin-fixed, paraffin-embedded, sectioned tissues (4 μm thick) were immunostained using the Labelled Streptavidin Biotin 2 System (BioGenex; San Ramon, CA, USA). Following peroxidase blocking with 0.3% H_2_O_2_/methanol for 30 min, specimens were blocked with phosphate-buffered saline (PBS) containing 5% normal horse serum (Vector Laboratories Inc., Burlingame, CA, USA). All incubations with mouse anti-human CLIC1 monoclonal antibody (1:175 dilution, Abcam,Cambridge, UK) were carried out overnight at 4°C. The specificity of this primary antibody has been demonstrated in previous studies of Wang et al. [11]. Then the specimens were briefly washed in PBS and incubated at room temperature with the anti-mouse antibody and avidin-biotin peroxidase (Vector Laboratories Inc., Burlingame, CA, USA). The specimens were then washed in PBS and color-developed by diaminobenzidine solution (Dako Corporation, Carpinteria, CA, USA). After washing with water, specimens were counterstained with Meyer’s hematoxylin (Sigma Chemical Co., St Louis, MO, USA). Nonneoplastic brain tissues were used as control tissues and non-immune IgG was also used as negative control antibody for immunohistochemical staining.

Assessment of immunohistochemical staining was evaluated by two independent pathologists. The scores of the two pathologists were compared and any discrepant scores were trained through re-examining the stainings by both pathologists to achieve a consensus score. The number of positive-staining cells showing immunoreactivity in cytoplasm for CLIC1 in ten representative microscopic fields was counted and the percentage of positive cells was calculated. The percentage scoring of immunoreactive tumor cells was as follows: 0 (0%), 1 (1–10%), 2 (11–50%) and 3 (>50%). The staining intensity was visually scored and stratified as follows: 0 (negative), 1 (weak), 2 (moderate) and 3 (strong). A final immunoreactivity scores (IRS) was obtained for each case by multiplying the percentage and the intensity score. Protein expression levels were further analyzed by classifying IRS values as low (based on a IRS value less than 5) and as high (based on a IRS value greater than 5).

### Real-time quantitative RT-PCR

The mRNA expression of CLIC1 in glioma and non-neoplastic brain tissues was detected by real-time quantitative RT-PCR analysis according to the conventional protocols of Tangdu hospital [18]. Especially, the primers were designed as follows: for human CLIC1, forward primer, 5′- ATG GCT GAA GAA CAA CCG -3′, and reverse primer, 5′- TTA TTT GAG GGA CTT TGA -3′; for human glyceraldehyde 3-phosphate dehydrogenase (GAPDH), forward primer, 5′- CCC ACT CCT CCA CCT TTG AC-3′, and reverse primer, 5′-ATG AGG TCC ACC ACC CTG TT-3′. Each sample was examined in triplicate and the amounts of the PCR products produced were non-neoplasticized to GAPDH which served as internal control.

### Statistical analysis

All computations were carried out using the software of SPSS version13.0 for Windows (SPSS Inc, IL, USA). Data were expressed as means±standard deviation (SD). The analysis of variance (ANOVA) was used to determine the statistical differences among the groups. A life table was calculated according to the Kaplan-Meier method. Hazard ratios for the time-to-event endpoint were estimated using the multivariate Cox regression analysis in a forward stepwise method to evaluate the effect of multiple independent prognostic factors on survival outcome. Differences were considered statistically significant when *p* was less than 0.05.

## Results

### CLIC1 mRNA expression in human glioma tissues

**Figure 1:**
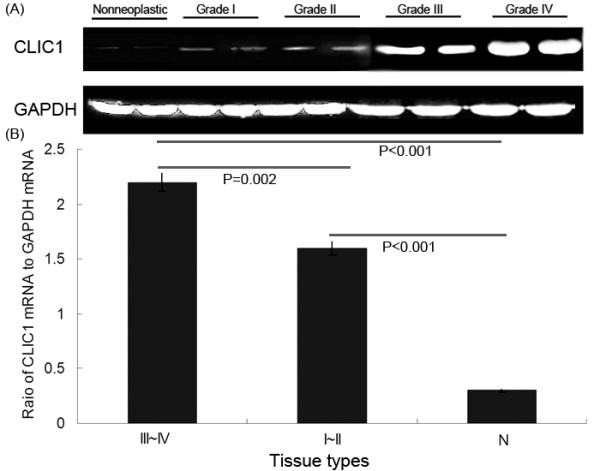
**CLIC1 mRNA expression in 20 glioma tissues with different grades and in non-neoplastic brain tissues were detected by real-time quantitative RT-PCR assay.** (**A**) Expression levels of CLIC1 mRNA in glioma tissues with different grades and non-neoplastic brain tissues. (**B**) A graphical representation of the CLIC1 mRNA level expression profiles in (A). ‘N’ refers to non-neoplastic brain tissues; ‘I~II’ refers to glioma tissues with grade I~II; ‘III~IV’ refers to glioma tissues with grade III~IV.

The expression levels of CLIC1 mRNA were detected in 20 glioma and 10 non-neoplastic brain tissues normalized to GAPDH. As shown in Figure 1A, the expression levels of CLIC1 mRNA were found to be distinctly increased in glioma tissues compared to non-neoplastic brain tissues, corresponding to the glioma WHO grades. The statistic results (Figure 1B) showed that its expression in high-grade (III-IV; 2.2±0.08) and low-grade (I-II; 1.6±0.06) gliomas were both significantly higher than that in non-neoplastic brains tissues (0.3±0.01; both P<0.001). Additionally, there was also a significant difference in mRNA copies of CLIC1 between high-grade (III-IV) and low -grade (I-II) glioma tissue specimens (P=0.002).

### Elevated expression of CLIC1 protein in human glioma tissues

**Figure 2:**
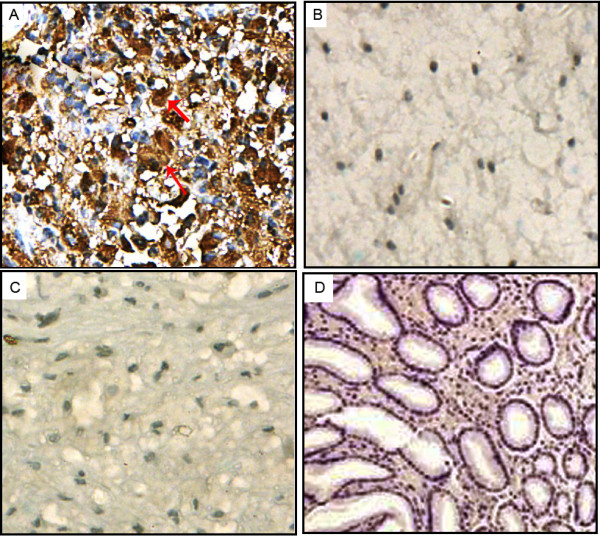
**Representative photographs for CLIC1 immunostaings in glioma tissues with WHO grade IV (A, Original magnification×400) and nonneoplastic brain tissues (B, Original magnification×400).** In glioma tissues, the immunostaining of CLIC1 was mainly expressed in the cytoplasm of tumor cells with brown yellow (marked by arrows). In contrast, Negative immunostaining was shown in the non-neoplastic brain tissues. Additionally, CLIC1 was not present in negative controls with non-immune IgG (Figure 1**C**, Original magnification×400) and in normal gastric tissues (Figure 1**D**, Original magnification×200).

The expression of CLIC1 protein was detected in 128 glioma specimens and 10 nonneoplastic brain tissues using immunohistochemical staining. Representative photographs of CLIC1 immunostainings were shown in Figure 2. In the glioma sections, CLIC1 was mainly detected in the cytoplasm (Figure 2A), which was consistent with previous studies on other cancers [10-12]. In contrast, the non-neoplastic brain tissues expressed a trace amount of CLIC1 (Figure 2B). CLIC1 was not present in negative controls with non-immune IgG (Figure 2C) and in normal gastric tissues (Figure 2D).

Of the 128 patients with gliomas, the high expression of CLIC1 was detected in 69.5% (89/128) of patients. For WHO grade III and IV tumors, 79.2% (76/96) of cases highly expressed CLIC1. However, for grade I and grade II tumors, 40.6% (13/32) of cases highly expressed CLIC1. According to these results, increased expression of CLIC1 was found to be associated with the histopathologic grading of the gliomas.

### Association of CLIC1 expression with clinicopatholigcal features of gliomas

The associations of CLIC1 protein expression with the clinicopathological factors of the glioma patients were summarized in Table 2. The over-expression of CLIC1 was detected in high-grade glioma tissues compared with those in low-grade tissues, and increased with ascending tumor WHO grades (P=0.005, Table 2). The increased expression of CLIC1 protein was also significantly correlated with low Karnofsky performance score (KPS) (P=0.008, Table 2). No statistically significant associations of CLIC1 with age at diagnosis and gender of patients were found (both P>0.05, Table 2).

**Table 2:** Association of CLIC1 protein expression in human glioma tissues with different clinicopathological features

**Clinicopathological features**	**No. of cases**	**CLIC1 expression**		**P**
		**High (n, %)**	**Low (n, %)**	
**Age**				
<55	52	36 (69.2)	16 (30.8)	NS
≥55	76	53 (69.7)	23 (30.3)	
**Gender**				
Male	76	51 (67.1)	25 (32.9)	NS
Female	52	38 (73.1)	14 (26.9)	
**WHO grade**				
I	18	6 (33.3)	12 (66.7)	**0.005**
II	14	7 (50.0)	7 (50.0)	
III	38	26 (68.4)	12 (31.6)	
IV	58	50 (86.2)	8 (13.8)	
**KPS**				
<80	78	61 (78.2)	17 (21.8)	**0.008**
≥80	50	28 (56.0)	22 (44.0)	

### Association of CLIC1 expression with overall survival in patients with gliomas

**Figure 3:**
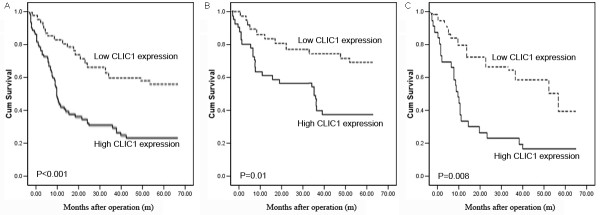
**Kaplan-Meier survival curves for glioma patients with high CLIC1 expression versus low CLIC1 expression.** (**A**) The cumulative 5-year overall survival was significantly shorter for patients with high CLIC1 expression than those with low CLIC1 expression (P<0.001). (**B** and **C**) Kaplan-Meier analysis showing the overall survival of glioma patients categorized according to the WHO grading criteria and status of CLIC1 expression. The cumulative 5-year overall survival was significantly different between high CLIC1 expression and low CLIC1 expression patients within subgroups of WHO Grades I~II (**B**, P=0.01) and III~IV (**C**, P=0.008).

Kaplan-Meier analysis using the log-rank test was performed to determine the association of CLIC1 expression with clinical outcome of glioma patients (Figure 3A). The results shown that high expression of CLIC1 was markedly associated with a shorter overall survival (P<0.001). During the follow-up period, 100 of 128 glioma patients (78.1%) had died. Of patients with high CLIC1 expression, 81 (81/89, 91.0%) were died; in contrast, 19 (19/39, 48.7%) of patients with low CLIC1 expression were died. The median survival time of patients with high CLIC1 expression (28.6 months, 95% confidence interval: 25.6–33.9) was significantly shorter than that of patients who had low CLIC1 expression level (50.1 months, 95% confidence interval: 41.2–58.6, P<0.001).

Nextly, the univariate analysis of individual variables revealed strong relationships between overall survival and WHO grade (P< 0.001), and CLIC1 expression (P<0.001). Additionally, the multivariate analysis identified CLIC1 expression (HR, 4.66; 95% CI, 2.31–10.29; P=0.01) and WHO grade (HR, 6.97; 95% CI, 2.12–12.46; P=0.008) as significant prognostic factors for glioma (Table 3).

**Table 3:** Cox multivariate analysis

**Parameter**	**Risk ratio**	**95% confidence interval**	**P**
**Age**	0.89	0.58–1.65	0.71
**Gender**	1.02	0.66–1.83	0.33
**WHO grade**	6.97	2.12–12.46	0.008
**KPS**	1.99	1.28–2.95	0.06
**Extent of resection**	1.29	0.89–2.13	0.11
**Type of adjuvant treatment**	1.37	1.02–2.24	0.11
**CCL20 expression**	4.66	2.31–10.29	0.01

Furthermore, we evaluated the prognostic significance of CLIC1 protein expression levels in different subgroups of glioma patients stratified according to the WHO grading. Notably, high CLIC1 expression also significantly correlated with shorter overall survival time in different glioma subgroups. Overall survival of glioma patients with high CLIC1 expression was significantly decreased than those with low CLIC1 expression in either Grades I~II subgroup (n=32; P=0.01; Figure 3B) or Grades III~IV subgroup (n=96; P=0.008; Figure 3C).

## Discussion

Similar with other human solid tumor cells, the glioma cells do not only have limitless replicative potential but also readily invade surrounding brain tissues and metastasize to other tissues, which make complete surgical resection practically impossible and lead to poor prognosis. Therefore, molecules involved in the aggressive process are potential prognostic and therapeutic markers. In the present study, our data shown for the first time that the up-regulation of CLIC1 at both mRNA and protein levels in glioma tissues compared with its expression in non-neoplastic brain tissues. Additionally, highly CLIC1 protein expression was significantly correlated with advanced WHO stage and low KPS scores, suggesting that this protein might be of clinical relevance in the aggressiveness of gliomas. Together with these findings, we also demonstrated that CLIC1 expression was a statistically significant risk factor affecting overall survival of patients with glioma and was an independent risk factor predicting short overall survival.

As a member of the CLIC family, CLIC1 functions as a real chloride channel in plasma and nuclear membranes [19]. The distribution of intracellular CLIC1 expression varies depending on the cell types, from intracellular vesicular pattern to intranuclear distribution. Its physiological roles include ion homeostasis modulation, cell volume regulation, transepithelial transport, and regulation of electrical excitability [20]. Accumulating number of studies have reported that CLIC1 is up-regulated in many tumor cells, such as hepatocellular carcinoma [10], gallbladder carcinoma [11], gastric cancer [12], colon cancer [13], nasopharyngeal carcinoma [21], and breast cancer [22], and plays important roles in tumor progression by modifying cell cycle, apoptosis, cell adhesion, and promoting tumor metastasis. For example, Chen et al. [12] found that the high levels of CLIC1 expression in gastric cancer significantly correlated with lymph node metastasis, lymphatic vessels and surrounding tissues infiltration, pathological staging, and survival time of patients; Wang et al. [23] shown that CLIC1 expression in lung adenocarcinoma was positively correlated with the T staging of the tumor and was negatively correlated with the shorter postoperative survival time of patients; Similarly, overexpression of CLIC1 was detected in gallbladder carcinoma and also found to significantly increase cell motility and invasion of the poorly metastatic gallbladder carcinoma cell line [11]. These results strongly imply that CLIC1 plays an important role in tumor advancement. However, its connection with human glioma has remained unknown. Our current study provided the evidence in support of such a connection using a cohort of 128 archived clinical glioma specimens. We first detected high expression of CLIC1 in glioma tissues compared with non-neoplastic brain tissues. Further support for a possible role of CLIC1 in glioma pathogenesis derived from the analysis that revealed a strong correlation of CLIC1 expression with the histopathological staging and inversely, with the survival of the disease. These findings are consistent with the previous reports which indicated that overexpression of CLIC1 is a potential prognostic marker for hepatocellular carcinoma [9], gallbladder carcinoma [10], gastric cancer [11], and lung adenocarcinoma [23].

In summary, our data provide the first evidence that CLIC1 expression might play an important role in the regulation of aggressiveness in human gliomas. The elevated expression of CLIC1 might represent a valuable prognostic marker for this disease. This study adds to the current realization on the involvement of CLIC1 in tumorigenesis and progression of human malignant tumors.

## Competing interests

The authors declared that they have no competing interests.

## Authors’ contributions

LW, SH, and YT carried out the Immunochemistry assay and drafted the manuscript. PJ and JZ carried out the pathological evaluation. LW, SH, YT, JZ, FF, and JZ participated in the survival analysis. YZ and G-dG conceived of the study, and participated in its design and coordination. All authors read and approved the final manuscript.
